# Automated Sperm Head Detection Using Intersecting Cortical Model Optimised by Particle Swarm Optimization

**DOI:** 10.1371/journal.pone.0162985

**Published:** 2016-09-15

**Authors:** Weng Chun Tan, Nor Ashidi Mat Isa

**Affiliations:** Imaging and Intelligent Systems Research Team (ISRT), School of Electrical and Electronic Engineering, Universiti Sains Malaysia, Nibong Tebal, Penang, Malaysia; Universite Blaise Pascal, FRANCE

## Abstract

In human sperm motility analysis, sperm segmentation plays an important role to determine the location of multiple sperms. To ensure an improved segmentation result, the Laplacian of Gaussian filter is implemented as a kernel in a pre-processing step before applying the image segmentation process to automatically segment and detect human spermatozoa. This study proposes an intersecting cortical model (ICM), which was derived from several visual cortex models, to segment the sperm head region. However, the proposed method suffered from parameter selection; thus, the ICM network is optimised using particle swarm optimization where feature mutual information is introduced as the new fitness function. The final results showed that the proposed method is more accurate and robust than four state-of-the-art segmentation methods. The proposed method resulted in rates of 98.14%, 98.82%, 86.46% and 99.81% in accuracy, sensitivity, specificity and precision, respectively, after testing with 1200 sperms. The proposed algorithm is expected to be implemented in analysing sperm motility because of the robustness and capability of this algorithm.

## Introduction

Image segmentation is a technique that aims to partition an image into multiple regions with similar attributes, such as colour and intensity [1]. This technique is an indispensable step in image processing because it extracts all important regions from an image and presents the image informatively. Image segmentation is one of the fundamental problems in computer vision [2]. Numerous studies that are related to image segmentation techniques, such as thresholding, fuzzy c-mean (FCM), region growing, split-and-merge and clustering, have been reported in the literature [1,3–6]. However, none of them are considerably accurate and precise, particularly in medical image segmentation.

This study uses human sperm sample images, which are captured under a phase-contrast microscope, to analyse human sperm motility. Apart from sperm concentration and sperm morphology, sperm motility is a crucial assessment to characterise male infertility [7]. Abnormal sperm is incapable of travelling long distance and penetrating the cervical mucus for fertilisation. The early discovery of the cause of male infertility provides an improved diagnosis for patients [8].

Generally, manual sperm analysis is often performed in a laboratory by andrologist experts. This method is claimed to be excessively reliant on the experience of technicians, procedures and human error. Therefore, computer assisted system analysis (CASA) systems have been introduced. CASA has become popular in sperm motility analysis because this system facilitates rapid assessments of sperm motility, velocity and other descriptors [9]. Many commercial and non-commercial versions of the CASA systems are presently available in the market. However, a study [10] stated that each of these systems has unique features that are suited for different research applications. Thus, different results are obtained when the same semen sample was tested using different systems. This issue has inspired researchers to propose considerably robust and accurate image processing techniques for sperm segmentation and classification.

Despite the many previous approaches, only multiple sperm cells segmentation methods are considered in this study. Park *et al*. [11] introduced a new method based on the Hough transform technique to segment the sperm head. The authors used morphological parameters, such as length, width, perimeter and area, in the aforementioned method. To achieve accurate results, the Hough transform technique assumed that the boundary of the sperm head was approximately equal to an ellipse. However, the part of the human sperm, particularly the sperm head, was distorted because of the rapid movement of the sperm. This method lacks accuracy and precision because the distorted sperm is not detected. Furthermore, Carrillo *et al*. [12] introduced an *nth-thresholding* method for sperm head segmentation. Firstly, the process started with the Otsu thresholding method to segment the bottom part of the head. Secondly, another fixed thresholding method is applied to segment the top part of the head. Thereafter, morphological operations are performed and a growing mask is built. These two processes tend to enhance the region of interest (ROI) and disregard other debris. Finally, the four regions, namely, of head, acrosome, nucleus and mid-piece, are shown as the final output image. Intuitively, this technique distinguishes normal cells from abnormal cells by using global thresholding and morphologic attributes. The selected value of the thresholding and morphological attribute parameters significantly affects the result. Notably, this method cannot perform in the presence of noise.

Another method of segmenting sperm cells was proposed by Abbiramy [13]. This method was performed using the Sobel edge detection to segment the sperm. Thereafter, the nine parameters, namely, head area, perimeter, head length, head width, midpiece length, tail length, orientation, eccentricity and equvidiameter, are used to extract the sperm head, mid-piece and tail. The values of these nine parameters are selected manually and empirically. The nine fixed parameters inhibit the detection of the distorted and agglutinated sperms. Compared with other parts of the human sperm, the detection of the human sperm head region is substantially meaningful in classifying sperm motility. Thus, the sperm head is selected as this study’s ROI.

The sperm segmentation algorithms showed good results in segmenting the sperm head. However, these algorithms still have the following drawbacks.

1. The distorted sperm shape cannot be detected accurately because this shape has been fixed in certain measurements (i.e. ellipse shape or morphological operation).
2. The agglutinated two or more sperms cannot be differentiated in a certain frame because of unexpected sperm combination.
3. The existing methods are sensitive to noise. In the worst case scenarios, image noise is misidentified as the sperm head.
4. The existing methods are not completely automated. These methods still require human intervention to initiate the sperm detection.

To solve the limitations of existing sperm segmentation techniques, the Intersecting Cortical Model (ICM) is proposed for implementation to the sperm image segmentation. The proposed method is capable of detecting the rotated or distorted moving sperms. The proposed method also designed to possibly achieve substantially high accuracy, sensitivity, specificity and precision results even with the existence of image noise. Furthermore, the proposed algorithm is specifically designed to differentiate the moving agglutinated sperms into multiple moving sperms. The proposed method eliminates the debris based on different intensity values of the sperm head and debris. The major concept of the proposed method is based on the intensity value of a pixel. A similar sperm feature based on the intensity value of an image is expected to be extracted based on the fired pulses of ICM. To ensure that the extracted sperm feature is similar, ICM links with neighbouring pixels to stimulate other neighbouring pixels to fire. This criterion ensures a considerably powerful ICM than those from simple thresholding techniques. The parameter values of ICM are often empirically determined via the repeated experiments. However, this parameter tuning strategy tends to restrict the potential of ICM. To alleviate this issue, Niu [14] proposed a self-adaptive parameter method on a modified ICM based on image characteristics. However, one of the important parameters, that is, number of iterations, was set constant (e.g. 20) and the setting may limit the segmentation ability of ICM. Unlike Niu’s approach, the current study attempts to determine the optimised iteration number that is required by the ICM model to solve each particular task. Each parameter with a different relationship was assigned with the static properties of each input image based on a previous study about the original model of ICM (i.e. PCNN) [15]. This method is determined inapplicable to the medical image (e.g. sperm image) because medical images contain non-uniform illumination areas that affect the static properties (e.g. highest intensity, lowest intensity and standard deviation) of the sample image. Furthermore, in a recent study [16], the PCNN parameters are assigned by modifying the fuzzy c-means method. This method increased the algorithm complexity and is unsuitable for sperm motility assessment. To date, no natural-inspired optimisation algorithms have been applied to address the parameter selection issue of ICM. New fitness function, which is feature mutual information, is proposed to facilitate the segmentation of the sperm features from real medical images. Thus, this study uses one of the most well-known nature-inspired metaheuristic search algorithms, namely, particle swarm optimization (PSO), to tune the ICM parameter settings. PSO is a popular meta-heuristic algorithm that uses the behaviours of a flock of birds in searching for a food source. In its original form, PSO consistently seeks to minimise or maximise a given function without requiring considerable assumptions on the function [17]. In addition in tuning parameters, the implementation of PSO into ICM (PSO–ICM) alters the proposed method from manual to automatic. According to [18], automatic segmentation is considerably desired, particularly in medical images.

The remainder of this paper is presented as follows. Section 2 provides a brief description on image pre-processing, ICM model, standard PSO description and feature extraction. Section 3 presents the data samples and performance of the proposed method by four main metrics. Section 4 evaluates the segmentation result via qualitative and quantitative analyses by comparing the proposed method with the other two sperm segmentation methods. Finally, Section 5 presents the overall conclusions.

## Materials and Methodology

### Sperm image acquisition

The study was approved by the Human Research Ethics Committee of Universiti Sains Malaysia with the reference code: USMKK/PPP/JEPeM[282.3(1)]. Human Research Ethics Committee of Universiti Sains Malaysia is listed under the Office for Human Research Protections (OHRP), United States Department of Health & Human Services. The Federal-wide Assurance (FWA) identification number is FWA00007718 and the Institutional Review Board (IRB) number is IRB00004494. The patients gave their written consent form before the sample was collected and the patients were made aware that their personal details are confidential throughout the research. All the semen samples used in this study were acquired from Hospital Universiti Sains Malaysia (HUSM). Patients were at least 15 years old at the time of sample collection. A total of 50 microliters of freshly ejaculated semen samples were used. Fresh semen samples were collected and stored in an incubator (37°C) before semen analysis. Sperm videos were recorded using 9.1-megapixel charge couple device (CCD) cameras under phase contrast microscopy. The recording time did not exceed 10 minutes to ensure that all sperms are active. The samples of human sperm images were captured using 40× magnification. The data set comprises 20 original images and ground truth images are used to test and evaluate the proposed algorithm. Each frame image consists of 60 sperm cells, thereby resulting in 1200 (i.e. 60 × 20) sperm cells that need to be segmented. This setup is supported by Chang [13]. Image size is set to 480 × 640 pixels, which is a resolution that is compatible with real medical image processing analyses, such as sperm infertility test. All the test images have been uploaded as supplement file S4 File.

### Methodology

**Fig 1:**
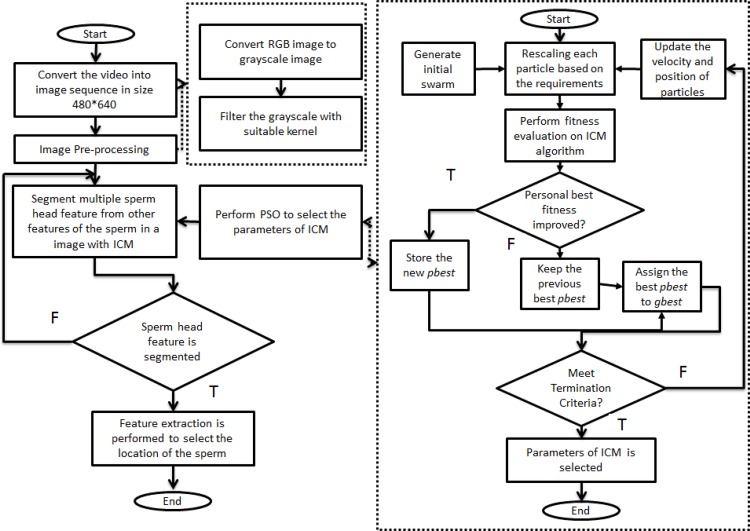
Flowchart of the proposed ICM–PSO method.

In this study, the overall process involved in developing an algorithm to segment and detect the human spermatozoa is illustrated by the flowchart shown in Fig 1. The input of the proposed method is a sperm video (see Fig 1). Initially, the sperm video will be converted into sequences of images that are 480 × 640 pixels in resolution. Image pre-processing is used to improve the quality of the sperm image because these images may be affected by non-uniform background and image noise. The pre-processing involves two stages. In the first stage, the images that are in RGB form are converted into grayscale images because such images consume less processing time as compared to colour images. In the second stage, the resultant grayscale image will be passed through a filter to emphasise the sperm head characteristic.

After pre-processing, the ICM model is proposed to segment the multiple sperm head features from other sperm heads. The ICM model is proposed because of its robustness and high accuracy, as presented in previous studies [19–21]. In addition, ICM is capable of solving the weakness occurred in sperm images, such as distorted sperm head, agglutinated sperm and image noise. Furthermore, PSO is implemented into the ICM model to perform parameter selection. Generally, initial swarms are generated in PSO. A scaling module is applied to each particle based on the requirement of ICM. This modification of PSO makes ICM fit in the PSO algorithm. Fitness evaluation based on the ICM model is performed on the particles. The fitness value is assigned to the global best fitness value if the current particle has the best fitness value. The previous global fitness value will be retained if the current particle has no improved fitness value. The process will continue until the termination criteria are achieved. In particular, all selected values are considered the optimum values when the termination criteria are achieved. The selection parameters are used in ICM to segment the sperm head feature. The final stage of the proposed algorithm involved is feature extraction. The centre of each segmented region is selected as the location of sperms because the nucleuses of sperms are our main target. This process is challenging because approximately 60 to 70 sperm nucleuses are needed to be extracted in each image frame.

#### Image pre-processing process

**Fig 2:**
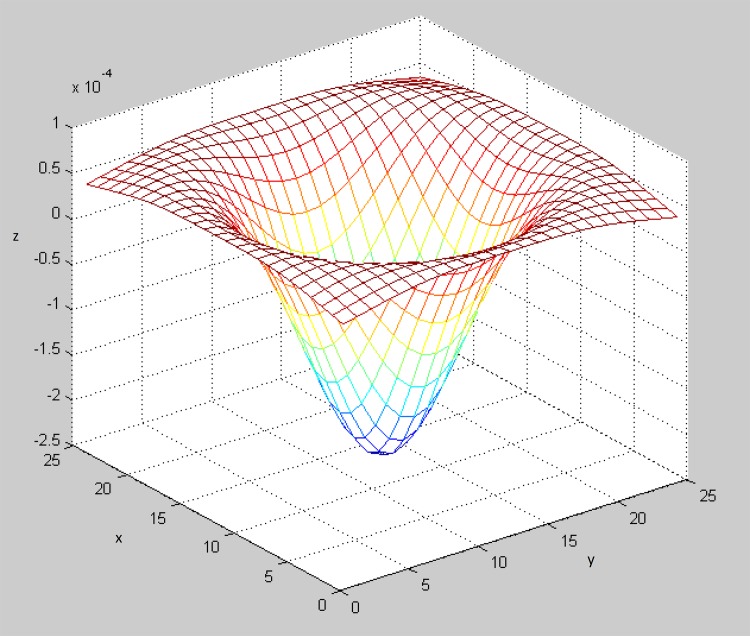
Laplacian of Gaussian Filter.

The first process involved in the proposed algorithm is image pre-processing. This process is implemented to transform the pixel value of the sperm head to reveal the sperm head characteristics. Thus, a kernel filter called the Laplacian of Gaussian (LoG) filter is used. The LoG filter is selected because it could produce a “Mexican hat” shape (see Fig 2). This shape, which is similar to the shape of a sperm head, is created when the LoG filter is implemented following Eq 1.

$$LoG(x,y)=-\frac{1}{\pi \sigma^{4}}\left[1-\frac{x^{2}+y^{2}}{2\sigma^{2}}\right]e^{-\frac{x^{2}+y^{2}}{2\sigma^{2}}}$$ (1)

where *σ* is a deterministic parameter in the LoG filter. Excessively large or small *σ* values could lead to the wrong detection of the sperm head region in the resultant image. When the LoG filter is convoluted with the segmented image, the latter will be scaled based on the LoG size.

#### Segmentation of the sperm head using ICM

In the succeeding process, ICM is used as the proposed segmentation technique to segment the head feature into the output image. ICM is a model that was developed to produce the same segmentation result as PCNN [22]. However, ICM has only a few parameters involved and this advantage allows it to have simpler and less complex network than PCNN. ICM minimises the cost of calculation and maintains the effectiveness of PCNN in image processing. The ICM model is considered the reduced version of the basic PCNN because the former comprises three coupled equations, whereas the latter comprises five coupled equations. Mathematically, ICM can be described as follows:

$$F_{i,j}[n+1]=f\;*\;F_{i,j}[n]+S_{i,j}+W\;{\left\{Y\right\}}_{i,j}$$ (2)

$$Y_{i,j}[n+1]=\{\begin{array}{cc} 1 & F_{i,j}[n]>E_{i,j}[n]\\ 0 & otherwise \end{array}$$ (3)

$$E_{i,j}[n+1]=gE_{i,j}[n]+hY_{i,j}[n+1]$$ (4)

where

- *Y*, output pulse (0 or 1)
- *F*, internal activation state
- *S*, normalised image pixels
- *n*, number of iteration
- *W*, synaptic weight matrix
- *f* and *g*, time decay coefficients
- *h*, amplitude coefficient of threshold
- *E*, dynamic threshold

Bijar [23] explained that the same sperm feature (i.e. sperm head) contains the same characteristics and one of them is intensity value. Thus, the proposed method attempts to use the external input (i.e. S_*i*,*j*_*)* of ICM as the main concept to segment the sperm head. In the proposed ICM method, the initial threshold value is set considerably high (i.e. value = 5) to produce an output image at the least iteration. The reasons behind this procedure are it saves time and makes ICM be more applicable to the real medical image particularly in infertility tests. W is the synaptic weight that is used to link with neighbouring neurons.

$$W=\left[\begin{array}{ccc} 0 & 0.01 & 0\\ 0.01 & 10 & 0.01\\ 0 & 0.01 & 0 \end{array}\right]$$ (5)

**Fig 3:**
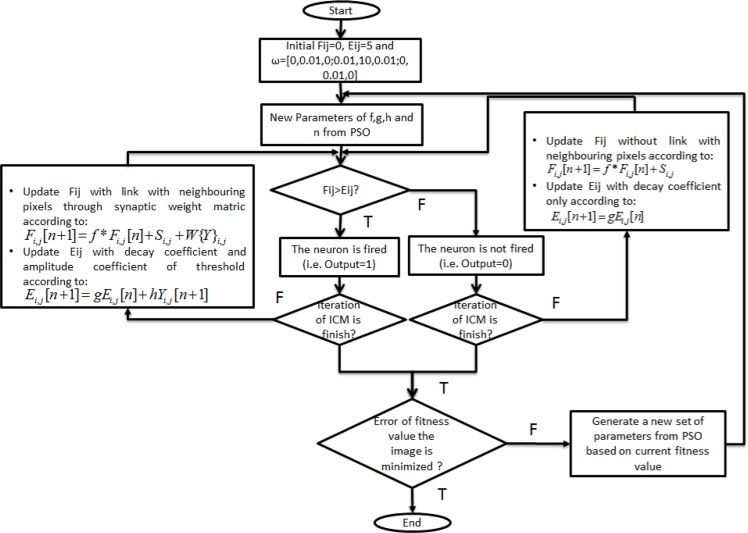
Flowchart of the modified ICM model.

Eq (5) shows that W is set as a constant matrix as recommended by Xiangyu [20] to achieve an improved focus or accuracy on segmented regions. Initially, stochastic parameters are generated from PSO to be implemented into the ICM model. Both feeding input (i.e. f*F_*i*,*j*_*[n]+S*_*i*,*j*_*[n]*) and linking input (i.e. *W{Y*_*i*,*j*_*}*) are connected to create the internal activation state *F*_*i*,*j*_. *F*_*i*,*j*_ is used to compare with a dynamic threshold *E*_*i*,*j*_ to produce output *Y*_*i*,*j*_. *Y*_*i*,*j*_ can be either 0 or 1. When *F*_*i*,*j*_ is less than *E*_*i*,*j*_, *Y*_*i*,*j*_ is set to 0. *F*_*i*,*j*_ and *E*_*i*,*j*_ are updated as equation, shown in Fig 3. Notably, *F*_*i*,*j*_ is updated without linking with the neighbouring pixels because the pixels have yet to be fired. In addition, *E*_*i*,*j*_ is decayed to a substantially small value for the next iteration. The iteration continues until *n* iteration. By contrast, when *F*_*i*,*j*_ is more than *E*_*i*,*j*_, *Y*_*i*,*j*_ is set to 1. *F*_*i*,*j*_ is updated by linking with the neighbouring pixels as the pixel is fired. The linking purpose is to stimulate the similar intensity value of the neighbouring pixel to be fired. Indirectly, the sperm head is extracted based on the salient feature (i.e. intensity value). Furthermore, *E*_*i*,*j*_ is updated with the decay coefficient (i.e. *g*) and amplitude coefficient of the threshold (i.e. *h*). The amplitude coefficient is consistently in a negative value to ensure that the neuron is always fired in the future. This step facilitates the extraction of the whole sperm head in the binary image.

After *n* iterations, the output binary image is evaluated via the fitness function in PSO to ensure that the sperm head is extracted. PSO will generate a set of parameters if the error of fitness value is not minimised. The entire process is repeated with the new set of parameters until the fitness value is met. Both time decay coefficient (*f* and *g*) must be set below 1 [19]. To ensure that the neurons will fire at least once, *f* should be set larger than *g* [19]. This condition must be obeyed to ensure the ICM operation.

Generally, when ICM is applied to the sperm images segmentation, 307200 neurons (i.e. subject to image size) will simultaneously undergo iteration. The input value after image pre-processing is taken as the external input of the neurons. Thus, the pixel neurons with considerably high intensity value will fire first. The fired neurons will affect the neighbouring neurons to fire in advance through the linking input. Subsequently, neurons with similar intensity value burst; this mechanism ensures the extraction of similar sperm features (i.e. sperm head). In this study, the background of the image will fire first because the background pixels have high intensity values. This process does not affect the segmentation ability of ICM.

Previously, the four parameters, namely, *f*, *g*, *h* and *n*, are selected manually to execute the ICM algorithm. However, this manual parameter setting approach limits the segmentation ability of ICM. The previous studies of [24] and [25] indicated that a proper value of parameter *n* was focused in selecting the right brain mask for brain extraction purpose. Notably, parameter *n* is a crucial parameter in the ICM module. This case motivates the authors to include this parameter in the current study. To ensure the applicability of ICM in any image, PSO is performed to optimise the four parameters. To the best of our knowledge, no natural-inspired optimisation algorithms have been applied to perform the parameter selection of ICM.

#### Optimised ICM parameters using PSO

In this study, ICM is implemented together with PSO to improve the accuracy of the algorithm by optimising all the parameters. Indirectly, this process automates the algorithm and avoids the parameter values from being selected empirically. The development of PSO is motivated by the collective and collaborative behaviours of birds flocking and fish schooling in searching of food [26]. PSO has elicited considerable attention in the research arena of computational intelligence because of its effectiveness and simple implementation in solving optimisation problems.

The PSO algorithm starts by randomly generating the particle’s position vector *x*_*i*,*d*_*(t)* and the particle’s velocity vector *v*_*i*,*d*_*(t)*. With *n* particles, each particle in the *d* dimensional search space tracks its position, which is associated with the best solution (i.e. fitness value) that it has achieved so far. This personal best position vector is called *pbest*. When a particle considers all the population as its neighbourhood best solution (i.e. fitness value), this group best position vector is called *gbest*. The particle position is subsequently computed based on the updated velocity vector as follows:

$$v_{i,d}(t+1)=\omega \;*\;v_{i,d}(t)+c_{1}r_{1,d}(pbest_{i,d}(t)-x_{i,d}(t))+c_{2}r_{2,d}(gbest_{d}(t)-x_{i,d}(t))$$ (6)

$$x_{i,d}(t+1)=x_{i,d}(t)+v_{i,d}(t+1)$$ (7)

where *i* is the current particle in the population of particle swarm, *d* is the dimension, *t* is the iteration number, *ω* is the inertia, *c*_*1*_ and *c*_*2*_ are the acceleration coefficients, *r*_*1*,*d*_ and *r*_*2*,*d*_ are random numbers in the range of [0,1], *v*_*i*,*d*_*(t)* is the *d*th dimension of the velocity vector *v*_*i*_ at the current iteration, *x*_*i*,*d*_*(t)* is the *d*th dimension of the position vector *x*_*i*_ at current iteration, *pbest*_*i*,*d*_ is the personal best position of the *i*th particle up to the current iteration (i.e. in self-cognitive component) and *gbest*_*d*_*(t)* is the global best position up to the current position (i.e. in social component) [27].

Generally, *c*_*1*_ and *c*_*2*_ are set as 2 in PSO. In addition, five crucial parameters need to be determined in PSO, namely, fitness function, population size (i.e. *n*), inertia factor (i.e. *ω*), dimension of the particle (i.e. *d*) and terminal condition.

Fitness function plays an important role in determining PSO performance. This function measures the judgment of the particle and guides the particle to move from the initial position to final position (i.e. the best position). Xu et al. [28] proposed that the entropy of the image as the fitness function. However, information entropy emphasised only the object and background regions of the segmented image without completely considering the original image [29]. Thus, information entropy is not applicable to many images, particularly medical images, because many of them lack of ground truth image. Any fitness function that is related to ground truth image will not be considered because the performance evaluation in the following section will compare the segmented image with the ground truth image. Thus, mutual information is proposed in the current study. Mutual information is an evolution of entropy as it involves the original intensity and segmented images. The latest study by Hage’s study [30], which proposed feature entropy to extract multiple bone features, has inspired the current study to create a new fitness function called feature mutual information. The selection of the feature mutual information is conducted via the heuristic approach to extract the sperm feature in the sperm image. In particular, mutual information is selected to be equal to 0.07 for all sperm images. This value is obtained by comparing the ground truth and original intensity images.

MI=H(X)+H(Y)-H(X,Y) (8)

where *H(X)* and *H(Y)* are the marginal entropy of the original intensity and segmented images, respectively, and *H(X*,*Y)* represents their joint entropy [31]. *H(X)*, *H(Y)* and *H(X*,*Y)* are defined as follows:

$$\text{H}(\text{X})=\text{-}\sum_{\text{x}}\rho_{\text{X}}(x)\log_{2}p_{X}(x)$$ (9)

$$\text{H}(\text{Y})=\text{-}\sum_{\text{y}}\rho_{\text{Y}}(y)\log_{2}p_{Y}(y)$$ (10)

$$\text{H}(\text{X},\text{Y})=\text{-}\sum_{\text{x},\text{y}}\rho_{\text{X},\text{Y}}(x,y)\log_{2}p_{X,Y}(x,y)$$ (11)

where *ρ*_*X*,*Y*_*(x*,*y)* is the joint probability density distribution function of image *X* and its segmented image *Y* and *ρ*_*X*_*(x)* and *ρ*_*Y*_*(y)* are the marginal probability distributions of *X* and *Y*, respectively.

Population size is the total number of particles that exist in the search space. An appropriate population size is needed to ensure the robustness and simplicity of PSO. A large population size deteriorates the particle’s convergence speed but results in high robustness to the system. By contrast, low population size may reduce the algorithm’s complexity but it tends to deliver local optimal results. The population size in the current study is set to 50 [32].

The objective of the inertia factor is to strike a balance between the exploitation and exploration searches of a particle in the solution search space. Exploitation is beneficial in fine-tuning the optimal solution, whereas exploration enables the particles to wander around the unvisited regions of search space. Notably, a large inertia value encourages exploration, whereas a small inertia value favours exploitation. Mathematically, the inertia factor can be defined as follows:

$$\omega =0.9-\frac{0.5\;*\;CurrentIteration}{MaxIteration}$$ (12)

The dimension of a particle is determined by the number of parameters need to be optimised. Four parameters are used, namely, the decay coefficients *f* and *g*, amplitude coefficient of threshold *h* and number of iterations *n*. The number of iterations has never been considered a crucial parameter because it is set constant in previous PCNN and ICM studies [14,28,33,34]. However, the number of iterations in ICM is important because our pilot study showed that the ICM model with the same *f*, *g* and *h* values could deliver different segmentation results when *n* is varied. PSO is stopped when the termination conditions are met. In the current study, the minimum value of fitness evaluation is selected as the terminal criterion.

**Fig 4:**
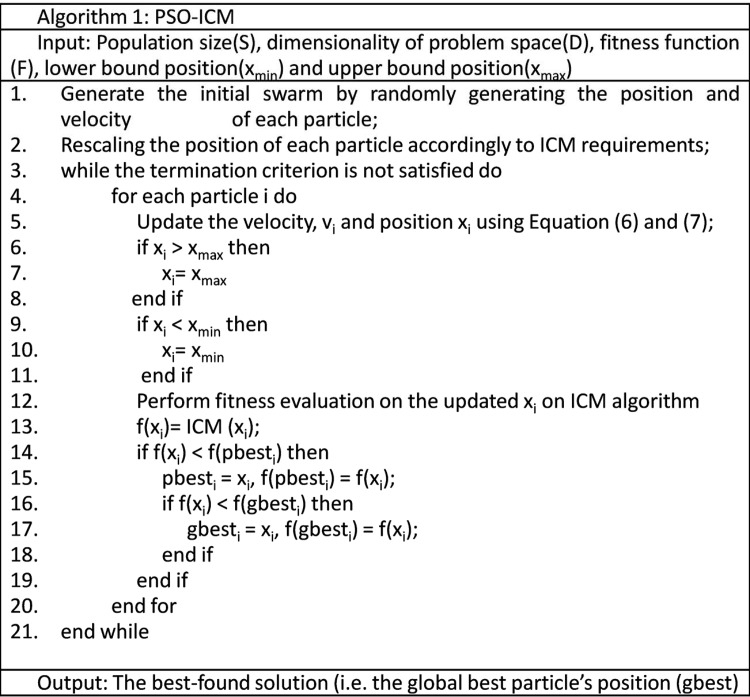
PSO–ICM algorithm.

**Fig 5:**
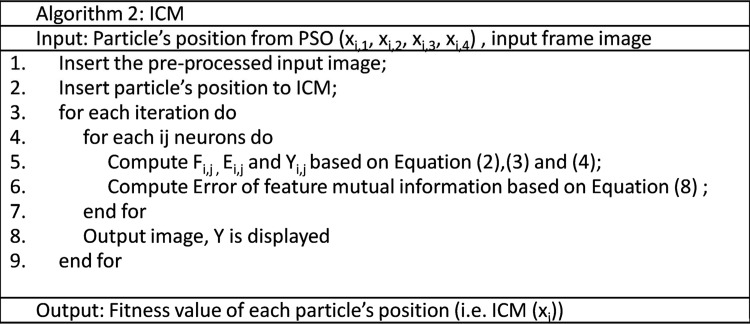
ICM algorithm.

The implementation of the PSO algorithm with all parameters is illustrated in Figs 4 and 5. The process starts with the random generation of the initial position of each particle (i.e. the four parameters *f*, *g*, *h* and *n* from ICM). Before fitness evaluation is performed on the particle, a scaling module is employed to each particle based on the ICM requirements. For example, *f* and *g* are scaled to 0 to 1, *h* is scaled to be negative and *n* is scaled to 5. The reason behind the modification of parameters *f* and *g* is to fit the ICM model to the PSO algorithm. To ensure that the fired neuron is pulsed in the following iterations, the value of *h* is fixed negative to maintain the activation energy at a lower value than the threshold value. This process simplified our algorithm to extract the sperm head feature once the neuron is fired. In addition, the modification of parameter *n* guided PSO to determine the optimal parameters in a lesser iteration without deteriorating the searching ability of PSO. Indirectly, this situation makes the method considerably applicable to real medical images (e.g. sperm image).

The process continues with fitness evaluation on the ICM algorithm via Eqs (2), (3) and (4); the fitness values (i.e. error of feature mutual information) are obtained for each particle position. The obtained fitness value *f(x*_*i*_*)* is compared with *pbest* and *gbest*. The aim of this process is to determine the minimum error from the feature mutual information. If f(x) is less than f(*pbest*) and f(*gbest)*, then the particle best position (i.e. *pbest)* and global best position (i.e. *gbest)* are replaced by the current particle position x. If f(x) is more than f(*pbest*) and f(*gbest)*, then the particle best position (i.e. *pbest)* and global best position (i.e. *gbest)* remain in the previous position. Thereafter, the velocity and position of the particle is updated with Eqs (6) and (7). The preceding process is repeated until the terminal condition is met. The targeted sperm head region should be extracted based on the obtained global best position (i.e. *gbest)*. The obtained global best position (i.e. *gbest)* is evaluated through quantitative and qualitative analyses in the following section.

#### Feature extraction

In this section, the segmented image is further analysed for feature extraction. The middle point of the segmented region should be selected as the sperm’s nucleus location because the target is the nucleus of the sperm. The segmented image has multiple segmented regions, thereby resulting in many challenges in detecting the nucleus. In this case, the segmented regions that are located near the image border are selected to be neglected to prevent incorrect detection. Each segmented region must be labelled to ensure the location of the sperm’s nucleus. Thereafter, the image size of each labelled region pixel is calculated. The mean of the horizontal and vertical axes is counted as the centroid of the sperm head. This process is simple and easily implemented.

### Performance Evaluation

**Fig 6:**
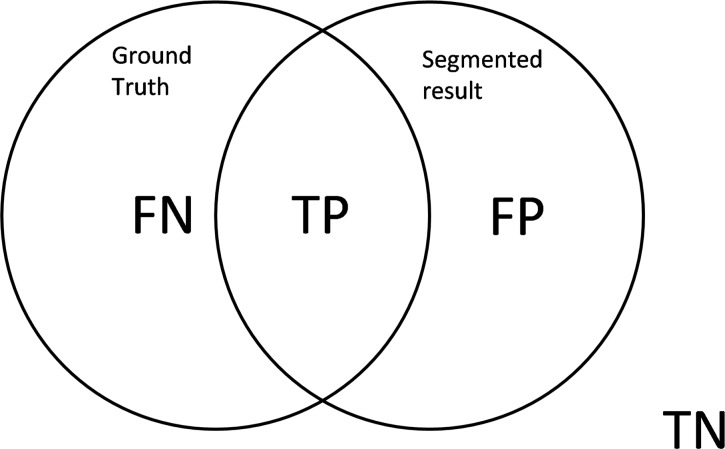
Four basic metrics to evaluate the performance between the segmented and ground truth images by expert [36].

To evaluate the performance of the proposed method, four main quantitative evaluations (i.e. accuracy, sensitivity, specificity and precision) are used. These evaluations are selected based on the recommendation of previous studies [12,30,35]. The evaluations are fused by four metrics: true positive (TP), true negative (TN), false positive (FP) and false negative (FN) (see Fig 6).

The current study defines TP as the segmented pixels that are correctly identified. FN occurs when the sperm pixels that should have been segmented are not observed in the segmented region. That is, FN reflects the amount of under-segmentation error. By contrast, FP occurs when pixels are wrongly segmented as sperm pixels. FP reflects the amount of over-segmentation error. Finally, TN occurs when the pixels that are not supposed to be segmented are correctly rejected by the algorithm. These four metrics are used to assess the accuracy, sensitivity, specificity and precision of the proposed algorithm.

Accuracy is commonly used to correctly compute the detection of the human sperm’s head, as well as represents the percentage of true results (both TP and TN) in the overall metrics examined. Accuracy can be expressed mathematically as follows:

$$\text{Accuracy}=\frac{TP+TN}{TP+TN+FP+FN}$$ (13)

Sensitivity, which is also known as TP rate, refers to the test’s ability to perfectly detect the region of the sperm head. High sensitivity rate is constantly desirable because it ensures the system’s output consistency with the prediction of medical experts (i.e. the proposed system is correctly identified as motile sperm in agreement with andrology physician).

$$\text{Sensitivity}=\frac{TP}{TP+FN}$$ (14)

Specificity refers to the capability of the algorithm to detect the targeted regions. High specificity indicates that the system is able to disperse between the human sperm head and debris. Mathematically, specificity is expressed as follows:

$$\text{Specificity}=\frac{TN}{TN+FP}$$ (15)

Precision, which is also known as positive predictive value (PPV), is defined as the proportional of the identified regions with respect to all positive regions present in the segment. This parameter is important to evaluate segmentation performance. Precision is different from accuracy because the former only measures true positive against all positives regions present. High accuracy does not mean the system has high precision and vice versa.

$$\text{Precision}=\frac{TP}{TP+FP}$$ (16)

This study also analysed computational time for each tested method. This analysis is used to test the complexity of those methods in terms of their implementation in sperm head detection. A short computation time shows lesser complexity in the implementation of the technique. All techniques are executed using MATLAB 8 on a personal computer with an Intel Core i7 3.4 GHz CPU with 4 GB of RAM.

In addition to quantitative analysis, qualitative analysis is also considered to track the visual difference of the proposed method. Qualitative analysis represents the capability of the proposed method to segment the sperm head and eliminate the unwanted regions (i.e. debris) that are are observed using the naked eye.

## Result and Discussions

In this section, the proposed method is compared with four state-of-the-art methods, namely, Abbiramy’s method [13], Chang’s method [37], ICM method without PSO optimisation and Carrillo [12]. Reference [37] reported that Carrillo’s method was commonly selected by other researchers as the state-of-the-art method for performance comparison. These four methods are selected for comparison because they showed the capability to detect multiple sperms (i.e. over 10 sperms), which is considerably applicable to the real sperm motility assessment images.

**Fig 7:**
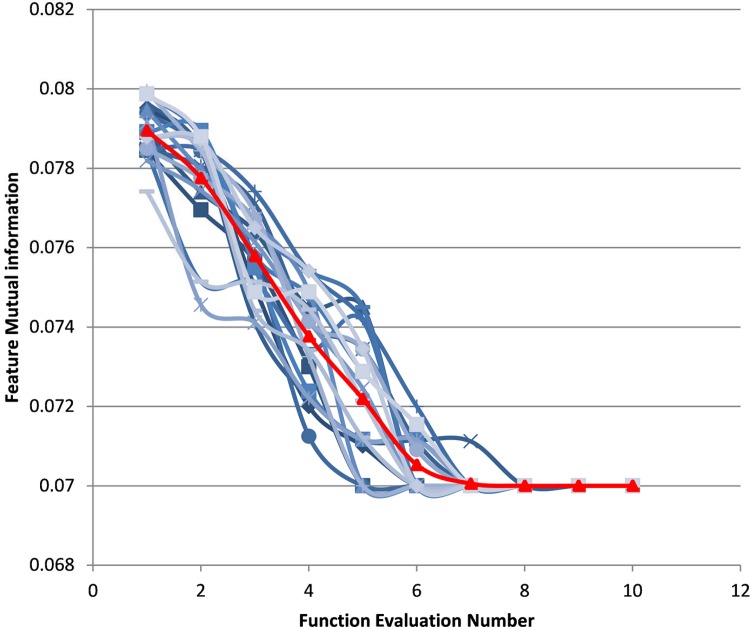
Convergence curve of the 20 sperm images.

Prior to the comparison, an analysis is performed to prove the reliability of the value set for the parameters of ICM (as mentioned in Section 3). The evaluation is conducted based on the convergence curve analysis for all 20 sperm images used. The results are illustrated in Fig 7. This figure shows that the termination condition (i.e. feature mutual information = 0.07) is met when the evaluation number is 10 or below for all 20 sperm images. The average value of the feature mutual information, which indicated the average fitness value of 20 sperm images, is shown in red. Given that the average fitness value is equal to 0.07 (as explained in Section 3), this result proves that the selected parameters in the 20 sperm images are successfully optimised via the PSO algorithm.

**Fig 8:**
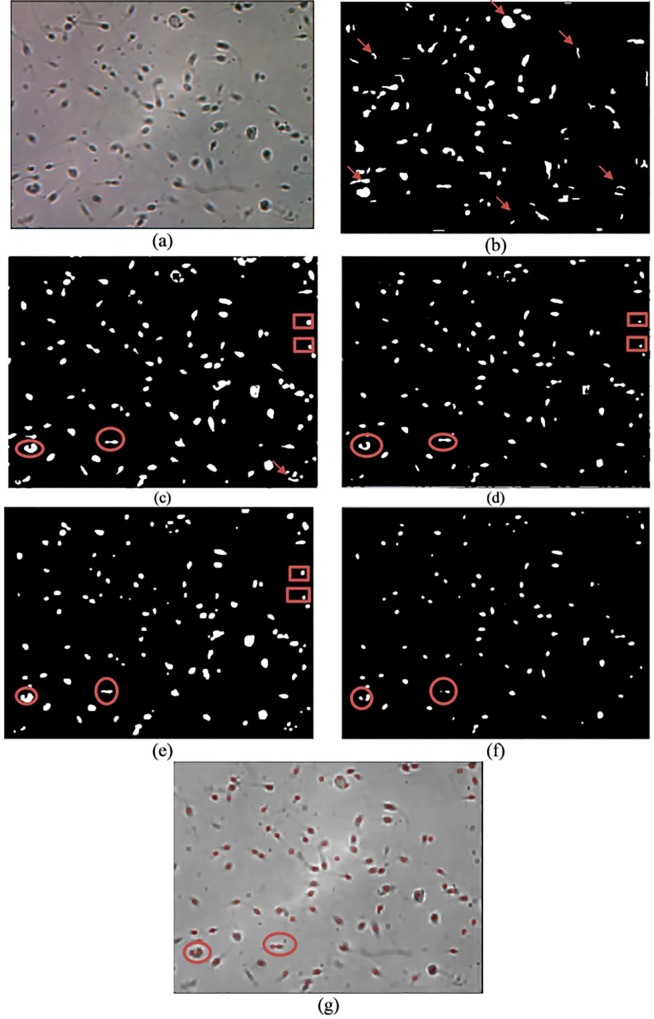
Resultant image for image frame 1 after the application of the (a) original image, (b) Abbiramy’s method, (c) Chang’s method, (d) manual ICM, (e) Carrillo’s method, (f) proposed method and (g) final detection of the sperm heads by the proposed method.

**Fig 9:**
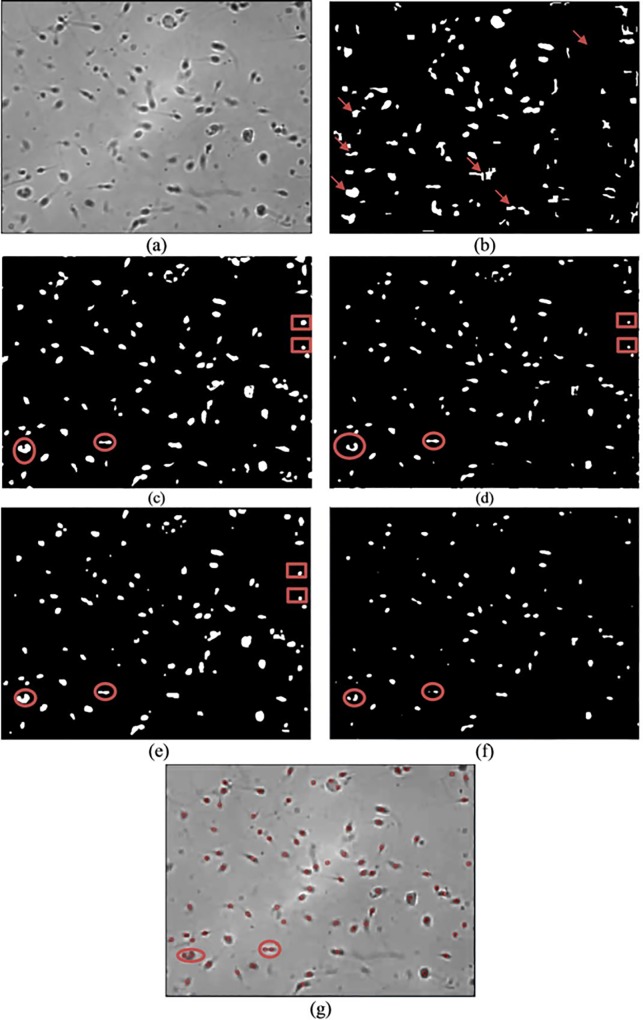
Resultant image for image frame 2 after the application of the (a) original image, (b) Abbiramy’s method, (c) Chang’s method, (d) manual ICM, (e) Carrillo’s method, (f) proposed method and (g) final detection of the sperm heads by the proposed method.

**Fig 10:**
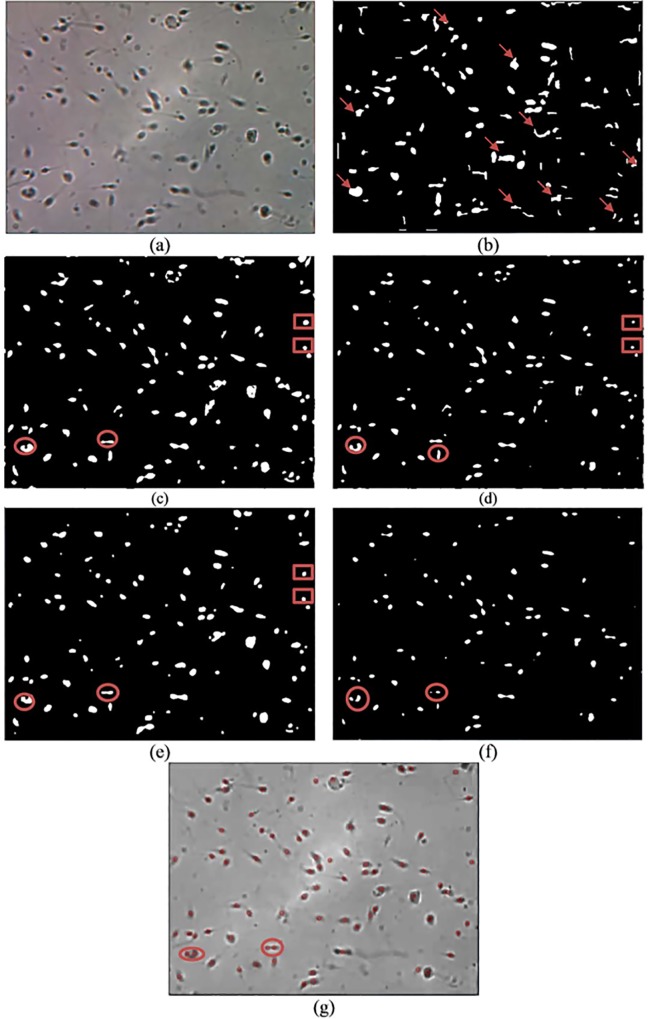
Resultant image for image frame 3 after the application of the (a) original image, (b) Abbiramy’s method, (c) Chang’s method, (d) manual ICM, (e) Carrillo’s method, (f) proposed method and (g) final detection of the sperm heads by the proposed method.

Qualitative and quantitative analyses are used for comparison analysis. The results of the qualitative analysis are shown in Figs 8–10, while the quantitative results are tabulated in Table 1. In Figs 8–10, image (a) represents the original image, whereas images (b), (c), (d), (e) and (f) are the resultant images that are produced after applying Abbiramy’s method, Chang’s method, ICM without PSO, Carrillo’s method and the proposed method, respectively. Image (g) represents the final detection of the sperm heads that are produced by the proposed method. Quantitative analysis is further performed by evaluating the performance of the proposed method. Table 1 tabulates the results. A good segmentation method should have accuracy, sensitivity, specificity and precision values of at least 0.8.

**Table 1:** Accuracy, sensitivity, specificity, precision and average computation time per image from Abbiramy’s method, Chang’s method, ICM without PSO method, Carrillo’s method and the proposed method for 20 images (i.e. 1200 sperm cells).

	Abbiramy’s method	Chang’s method	Manual ICM	Carrillo’s method	Proposed method
Accuracy	0.9745	0.9807	0.9689	0.9577	**0.9814**
Sensitivity	0.9838	0.4384	0.9856	0.9877	**0.9882**
Specificity	0.2711	**0.9815**	0.3413	0.8954	0.8646
Precision	0.9867	0.0297	0.9803	0.9585	**0.9981**
Average computational time per image (seconds)	3.6974	1.0564	**0.2160**	1.3343	0.7834

The sperm head is selected as the only targeted sperm feature because in the final stage of the study, the location of the sperm nucleus is the major concern in analysing sperm motility. Notably, the 20 sperm images suffered from non-uniform illumination and low contrast; they were also affected by unwanted noise. The non-uniform illumination is likely caused by uneven lighting conditions when images are captured. The Abbiramy’s method produced the worst results where many wrongly segmented regions are present, as shown by the arrows (see Figs 8(B), 9(B) and 10(B). Table 1 shows that this result is proven by a low specificity value (i.e. 0.2711). This result is mainly caused by the inability of Abbiramy’s method to detect the distorted or rotated sperms. Such sperms are mainly caused by the movement of the sperm in the depth of the chamber (i.e. above 20 microlitres based on the WHO manual) [32]. In addition, this finding proves that the proposed method is unaffected by the distorted or rotated sperms because of its high accuracy, sensitivity, specificity and precision. The reason is that the proposed method extracts the sperm head based on the intensity value of the sperm instead of geometrical characteristics that required prior knowledge on the sperm. Table 1 shows that Abbiramy’s method produced high accuracy (i.e. 0.9745) and precision (i.e. 0.9867) but the lowest specificity (i.e. 0.2711). This result proved that Abbiramy’s method tends to incorrectly segment many regions. In addition, Abbiramy’s method is affected by the rapid movement of the sperm. For example, debris with similar sizes of sperm head are incorrectly detected as sperm heads, as indicated by the marked arrows. By contrast, the proposed method showed no detection of the debris as sperm heads.

Chang’s method presented a better result than that of Abbiramy’s method (see Figs 8(C), 9(C) and 10(C). Compared with the original image, Chang’s method successfully segmented many sperm heads with only a few detected debris. However, the agglutinated sperms remain unable to be differentiated, as shown by the circled regions. The original image has two agglutinated sperms in both circled regions. However, in the resultant image, Chang’s method wrongly segmented them as one single sperm. This problem of Chang’s method has led to low sensitivity and precision values of 0.4384 and 0.0298, respectively. These values are substantially lower than those of the proposed method, which showed high sensitivity (i.e. 0.9882) and high precision (i.e. 0.9981).

To prove the importance of automatic parameter optimisation of ICM as introduced in the proposed method, performance comparison is also made with the conventional ICM method. In the conventional ICM method, the selected parameters are selected manually and experimentally. The results clearly indicate that the manual ICM failed to differentiate the sperms from the debris and agglutinated sperms. These phenomena can be observed in the rectangular and circled regions (see Figs 8(D), 9(D) and 10(D)). The observation is supported by the low values in all the metrics, particularly in specificity (i.e. 0.3413). This finding clearly proved that the introduction of the fitness function (i.e. feature mutual information) significantly improved the capability of the conventional ICM. Notably, the fitness function is designed specifically for sperm head segmentation.

Carrillo’s method produced comparable segmented results with the proposed method (see Figs 8(E), 9(E) and 10(E) and the results tabulated in Table 1). However, limitations are produced by this method. Similar to the previous methods, as shown from the rectangular and circled region in Figs 8(E), 9(E) and 10(E)), Carrillo’s method has misclassified the debris and agglutinated sperm heads as sperm heads and one sperm head region, respectively. These types of misclassifications result in low accuracy (i.e. 0.9577) and precision (i.e. 0.9585) by Carrillo’s method compared with the proposed method (see Table 1). With increment between 0.7% to 2.47% and 1.16% to 4.13% for accuracy and precision, respectively, compared with Carrillo’s method, the proposed method has successfully shown high capability in (1) detecting distorted sperm head, (2) differentiating between sperms and debris and (3) separating the agglutinated sperms. By contrast, the proposed method showed slightly lower specificity (i.e. 0.8646) compared with Carrillo’s method (i.e. 0.8954). The reason behind this is the proposed method is able to consider possible sperm heads even when they are visually unclear (e.g. rapid movement of sperm), thereby occasionally leading to misidentification of the sperm head regions as debris. However, this type of misidentification is hardly observed in the resultant images produced by the proposed method. This case is proven by the small difference in specificity between the proposed method and Carrillo’s method (i.e. a difference of merely 0.0308). In the latest article by Leeflang [33], accuracy is claimed to be a more clinically relevant measurement compared with specificity. Instead of one metric, this idea motivates us to emphasise the importance of high accuracy, sensitivity, specificity and precision in the proposed method. Particularly, the proposed method is the only method that is able to differentiate the agglutinated sperm.

As a comparison for time efficiency, Table 1 records and tabulates the average time acquired by all the tested methods to process sperm images. Table 1 shows that Abbiramy’s method has the longest computational time (i.e. 3.6974 s). This finding suggests that this method is not the ideal candidate for sperm motility assessment method. By contrast, the proposed method is ranked second with 0.7834 s of computational time after the manual ICM (i.e. 0.2160 s of computational time. Accordingly, the manual ICM method consumes the least computational time by a large margin. This finding possibly suggests that the ICM method is a good candidate for sperm motility assessment. However, the results presented in Table 1 have shown that the proposed method produced high performance, particularly in specificity (i.e. 0.8646 s for the proposed method compared with 0.3413 s for the manual ICM). Thus, by producing the best sperm detection performance and second least computational time, it is clear that the proposed method is the best choice to be used as sperm motility assessment method compared with other state-of-the-art methods.

**Fig 11:**
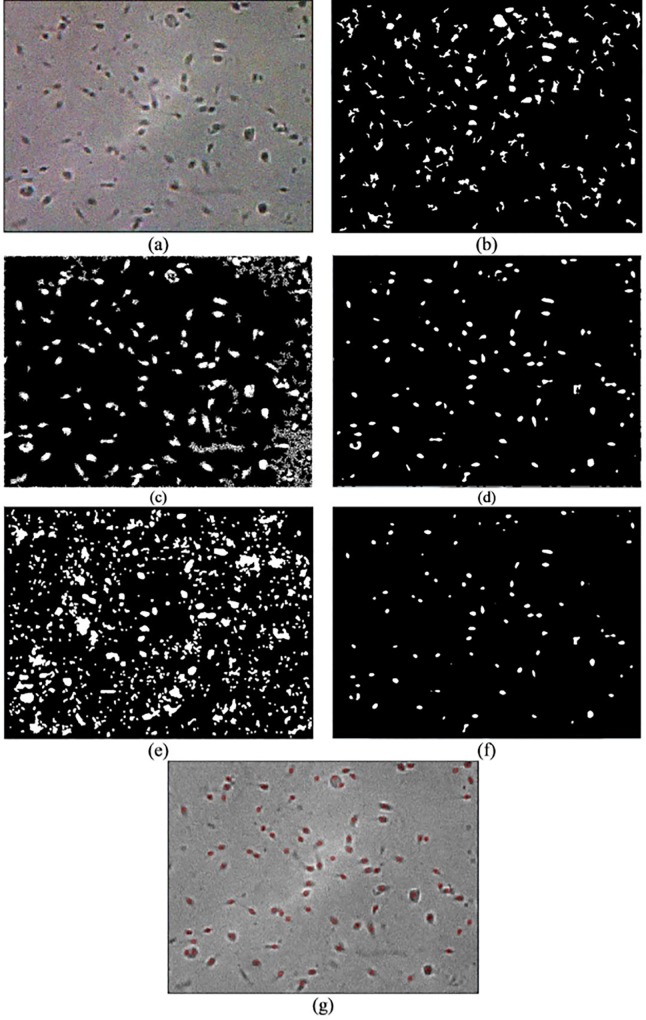
Resultant image of frame 1 with Gaussian noise after the (a) original image, (b) Abbiramy’s method, (c) Chang’s method, (d) manual ICM, (e) Carrillo’s method, (f) proposed method and (g) final detection of the sperm heads by the proposed method

**Fig 12:**
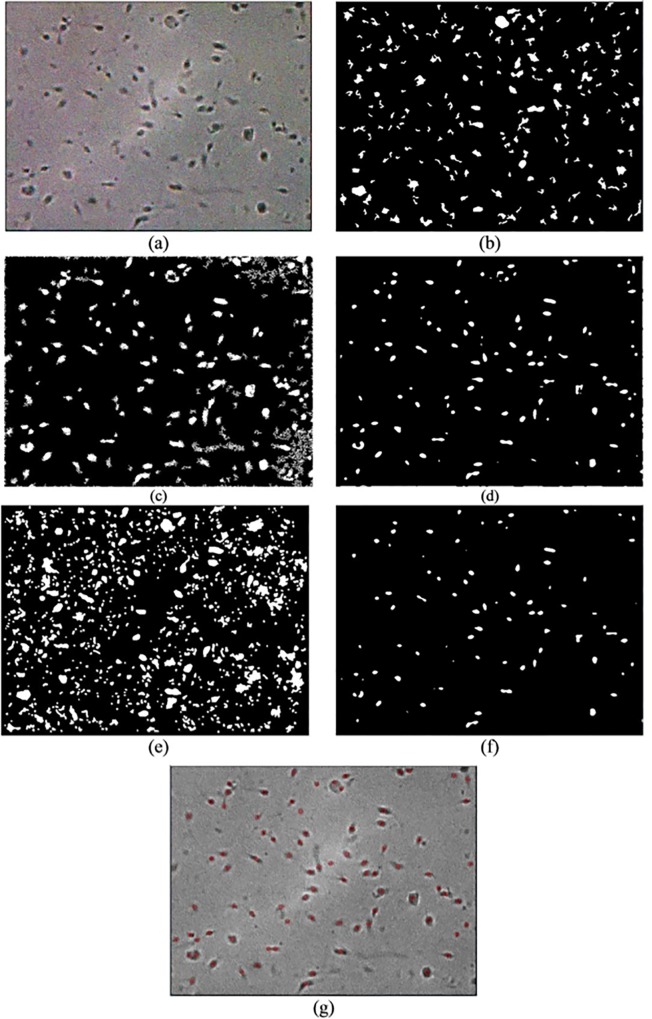
Resultant image of frame 2 with Gaussian noise after the (a) original image, (b) Abbiramy’s method, (c) Chang’s method, (d) manual ICM, (e) Carrillo’s method, (f) proposed method and (g) final detection of the sperm heads by the proposed method

**Fig 13:**
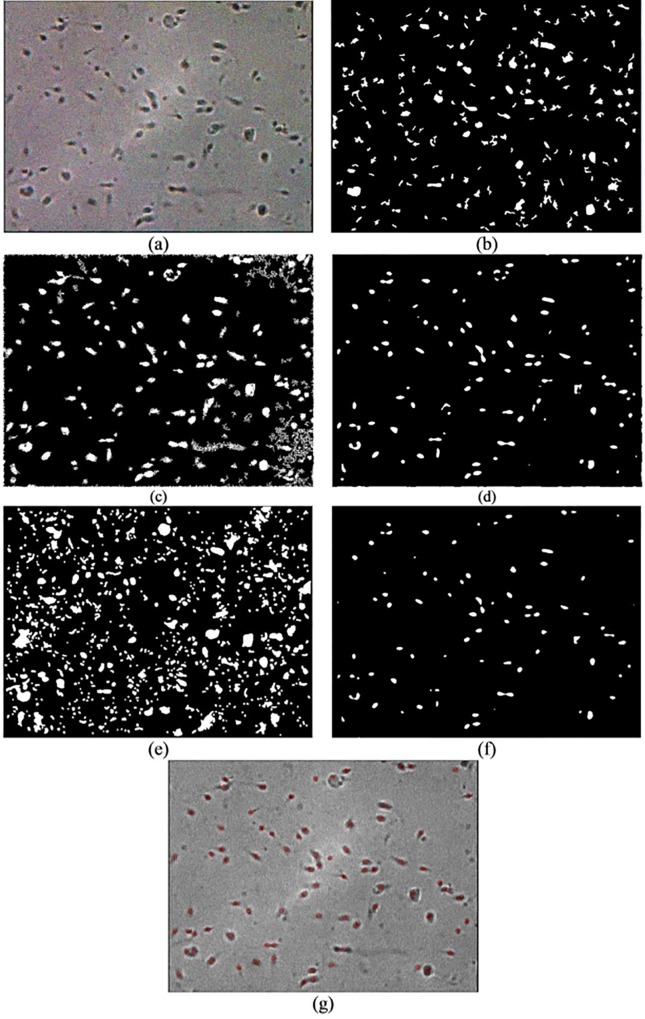
Resultant image of frame 3 with Gaussian noise after the (a) original image, (b) Abbiramy’s method, (c) Chang’s method, (d) manual ICM, (e) Carrillo’s method, (f) proposed method and (g) final detection of the sperm heads by the proposed method

To further evaluate the robustness of the proposed method against unwanted noise, sperm images that are affected by Gaussian noise are used as input images (see Figs 11–13). Overall, only the ICM method showed better robustness against noise compared with the other methods. Evidently, Abbiramy’s method, Chang’s method and Carrilo’s method are affected by Gaussian noise (see Figs 11–13). For example, Abbiramy’s method segmented many unusual sperm head shapes, Chang’s method presented many incorrect initial seeds that resulted in wrong segmentation result and Carrilo’s method showed poor segmentation results by detecting all the noise. Although the manual ICM method produced better results than the methods of Abbiramy, Chang and Carillo’s method (i.e. less prone to noise), misidentification of debris as sperm heads and misidentification of the two agglutinated sperm could still be observed in the resultant images. The proposed method has been proven as the best method among all the tested methods because the former does not produce the aforementioned types of misidentifications and is less prone to noise.

The results indicate that the proposed method showed its robustness because it produced high accuracy output for the 20 different non-uniform illumination and low contrast sperm images. These findings prove that combining the ICM model with the PSO algorithm has demonstrated the proposed method’s potential to achieve promising segmentation results, as verified by qualitative and quantitative analyses.

Overall, the proposed method automatically provides satisfactory segmentation results of sperm. However, a few limitations of the proposed method must still be addressed to apply the technique in routine clinical practice. Firstly, the lighting of the obtained image must be adjusted well to create a contrast between ROI and the background. The failure to create the contrast will affect the segmentation result. Secondly, the proposed method only focuses on producing good segmentation results in terms of high accuracy, sensitivity, specificity and precision. For future industry potential, computational time should be one of the focused topics.

## Conclusion

In this study, ICM is implemented for sperm image segmentation. The selection of this model is justified by the three main advantages offered, that is, high accuracy and precision, ability to segment the agglutinated sperms and immunity to image noise. The major difficulty faced by ICM is the selection of parameters that could significantly affect the final segmentation result. To address this issue, the PSO technique is used as an automated parameter tuning tool. Notably, this technique automates the system, as opposed to manual operation. The final segmentation result is compared with other sperm segmentation methods. The result shows that the proposed method has high accuracy, sensitivity, specificity and precision. In the future, this method is expected to be implemented in sperm motility analysis.

## Supporting Information

S1 File Human Ethic Approval. (PDF)

S2 File Consent Form. (PDF)

S3 File Data Collection Form. (PDF)

S4 File Dataset. (RAR)
