# Supplementary material for: Automated Sperm Head Detection Using Intersecting Cortical Model Optimised by Particle Swarm Optimization
Source: PLoS One. 2016 Sep 15;11(9):e0162985. doi: 10.1371/journal.pone.0162985 (PMC5025108; doi:10.1371/journal.pone.0162985)
Supplement: S1 File — (PDF) [file pone.0162985.s001.pdf]

UNIVERSITI SAINS MALAYSIA

Jawatankuasa Etika Penyelidikan Manusia USM (JEPeM)
Human Research Ethics Committee USM (HREC)

PUSAT PENGAJIAN KEJURUTERAAN ELEKTRIK & ELEKTRONIK
TERIMA
08 JUL 2014

Universiti Sains Malaysia
Kampus Kesihatan,
16150 Kubang Kerian,
Kelantan. Malaysia.
T: 609 - 767 3000 *samb.* 2354/2362
F: 609 - 767 2351
E: jepem@usm.my
www.jepem.kk.usm.my

Our. Ref. : USM/JEPeM/282.3.(1)
Date : 30th June 2014

Assoc. Prof. Dr. Nor Ashidi Mat Isa
School of Electrical & Electronic Engineering
Engineering Campus
Universiti Sains Malaysia
14300 Nibong Tebal, Pulau Pinang.

The Human Research Ethics Committee, Universiti Sains Malaysia (FWA Reg. No: 00007718; IRB Reg. No: 00004494) has approved in principle the study mentioned below:

**Title**	Development of Computational Intelligence Infertility System Based on Sperm Motility Analysis.		
**Protocol No**	-	**Principle Investigator**	Assoc. Prof. Dr. Nor Ashidi Mat Isa
**Date of approval**<br>Protocol received<br>Reviewed by Committee<br>Received Amended Protocol	30th June 2014<br>9th January 2014<br>23rd April 2014<br>23rd June 2014	**Co-Investigator(s)**	Dr. Mahaneem Mohamed<br>Tan Weng Chun<br>Khairunnisa Hasikin
**Research Center**	Hospital Universiti Sains Malaysia and Engineering Campus, Universiti Sains Malaysia.	**Date of study start**	July 2014 – June 2016
**Financial Support**	-	**Number of Samples**	110 subjects

The following item (✓) have been received and reviewed:-

(✓) **Ethical Approval Application Form**
(✓) **Research Proposal**
(✓) **Patient Information Sheet and Consent Form**
(✓) **Data Collection Form**

Investigator(s) are required to:

a) follow instructions, guidelines and requirements of the Human Research Ethics Committee, Universiti Sains Malaysia (JEPeM)
b) report any protocol deviations/violations to Human Research Ethics Committee (JEPeM)
c) comply with International Conference on Harmonization – Guidelines for Good Clinical Practice (ICH-GCP) and the Declaration of Helsinki
d) note that Human Research Ethics Committee (JEPeM) may audit the approved study.

PROFESSOR DR. HANS AMIN VAN ROSTENBERGHE
Chairperson
Human Research Ethics Committee
