# Supplementary material for: Automated Sperm Head Detection Using Intersecting Cortical Model Optimised by Particle Swarm Optimization
Source: PLoS One. 2016 Sep 15;11(9):e0162985. doi: 10.1371/journal.pone.0162985 (PMC5025108; doi:10.1371/journal.pone.0162985)
Supplement: S2 File — (PDF) [file pone.0162985.s002.pdf]

LAMPIRAN A

MAKLUMAT KAJIAN

Tajuk Kajian: Pembangunan Sistem Kemandulan Berkomputer Kepintaran Berdasarkan Analisis Sperma Motiliti

Nama Penyelidik: Prof. Madya Dr Nor Ashidi Mat Isa
Dr Mahaneem Mohamed (MMC: 31287)
Tan Weng Chun

PENGENALAN

Anda dipelawa untuk menyertai satu kajian penyelidikan secara sukarela yang melibatkan penggunaan air mani. Penyelidikan ini diperlukan untuk menbangunkan sistem kemandulan berkomputer kepintaran berdasarkan analisis sperma motiliti. Penyelidikan ini akan mengambil air mani yang berlebihan yang akan dibuang selepas analisa cecair semen yang biasa dijalankan di Makmal Sitologi USM. Pergerakan Sperma akan direkodkan dengan menggunakan video camera dan mikroskop. Sebelum anda bersetuju untuk menyertai kajian penyelidikan ini, adalah penting anda membaca dan memahami borang ini. Sekiranya anda menyertai kajian ini, anda akan menerima satu salinan borang ini untuk disimpan sebagai rekod anda.

Penyertaan anda di dalam kajian ini dijangka mengambil masa sehingga 4 minggu. Seramai 110 pesakit akan menyertai kajian ini.

TUJUAN KAJIAN

Kajian ini bertujuan untuk mengkaji masalah kemandulan dengan mengira motilitasi sperma melalui sistem berkomputer. Sistem berkomputer ini diramalkan akan memberi keputusan yang lebih tepat daripada cara manual.

Terdapat kemungkinan maklumat yang dikumpulkan semasa kajian ini akan dianalisa oleh pihak penyelidik pada masa depan untuk menilai sperma motilitas bagi kegunaan penyelidikan atau untuk tujuan perubatan atau saintifik lain yang selain dari yang kini dicadangkan.

KELAYAKAN PENYERTAAN

Penyelidik yang bertanggungjawab dalam kajian ini atau salah seorang kakitangan kajian telah membincangkan kelayakan untuk menyertai kajian ini dengan anda. Adalah penting anda berterus terang dengan doktor dan kakitangan tersebut tentang sejarah kesihatan anda. Anda tidak seharusnya menyertai kajian ini sekiranya anda tidak memenuhi semua syarat kelayakan.

Beberapa **keperluan** untuk menyertai kajian ini adalah –

- Anda telah disyaki mengalami masalah kemandulan .
- Anda mesti mengadakan pantang seksual sekurang-kurangnya 2 hingga 3 hari

PROSEDUR-PROSEDUR KAJIAN

Pada lawatan anda, sekiranya anda setuju menyertai kajian, anda perlu mendapatkan kebenaran daripada doktor untuk menjalani ujian kemandulan. Air mani akan didapatkan daripada anda dan hantar ke lab patologi untuk menjalankan ujian secara manual. Air mani yang selebihnya akan digunakan untuk menjalani kajian-kajian penyelidikan dengan merekodkan video untuk dianalisiskan pada masa hadapan.

RISIKO

Sekiranya anda menyertai kajian ini, anda mungkin mengalami rasa tidak selesa dan kesulitan semasa mengurus dengan kakitangan hospital. Tetapi, ingat apa yang dilakukan oleh anda adalah amat dihargai oleh pihak kita dan ia akan diperlukan untuk membantu dalam kajian masalah kemandulan. Hanya sample yang berlebihan daripada air mani yang dibekalkan akan digunakan untuk kajian. Tiada sample yang lebih perlu dibekalkan untuk tujuan kajian. Jika apa-apa maklumat penting yang baru dijumpai semasa kajian ini yang mungkin mengubah persetujuan anda untuk terus menyertai kajian ini, anda akan diberitahu secepat mungkin.

MELAPORKAN PENGALAMAN KESIHATAN

Jika anda mengalami apa-apa kecederaan, kesan buruk, atau apa-apa pengalaman kesihatan yang luarbiasa semasa kajian ini, pastikan anda memberitahu jururawat atau doktor yang membenarkan anda menjalani ujian kemandulan.

PENYERTAAN DALAM KAJIAN

Penyertaan anda dalam kajian ini adalah secara sukarela. Anda berhak menolak untuk menyertai kajian ini atau anda boleh menamatkan penyertaan anda pada bila-bila masa, tanpa sebarang hukuman atau kehilangan manfaat yang sepatutnya anda perolehi.

Penyertaan anda juga mungkin boleh diberhentikan oleh doktor yang terlibat dalam kajian ini tanpa persetujuan anda. Sekiranya anda berhenti menyertai kajian ini, doktor yang terlibat di dalam kajian ini atau salah seorang kakitangan akan berbincang dengan anda mengenai apa-apa isu perubatan berkenaan dengan pemberhentian penyertaan anda.

MANFAAT YANG MUNGKIN [Manfaat terhadap Individu, Masyarakat, Universiti]

Prosedur kajian ini akan diberikan kepada anda tanpa kos. Anda mungkin menerima maklumat tentang kesihatan anda daripada pemeriksaan fizikal dan ujian makmal yang dilakukan dalam kajian ini. Hasil atau maklumat kajian ini diharapkan, dapat memberi manfaat kepada pesakit-pesakit pada masa hadapan. Anda tidak akan menerima sebarang pampasan kerana menyertai kajian ini.

PERSOALAN

Sekiranya anda mempunyai sebarang soalan mengenai prosedur kajian ini atau hak-hak anda, sila hubungi;

<Dr. Mahaneem Mohamed> & <31287>
<Jabatan Fisiologi>
<Pusat Pengajian Sains Perubatan>
<USM Kampus Kesihatan>
<No Tel: 09-7676158>

Sekiranya anda mempunyai sebarang soalan berkaitan kelulusan Etika atau sebarang pertanyaan dan masalah berkaitan kajian ini, sila hubungi;

Puan Mazlita Zainal Abidin
Setiausaha Jawatankuasa Etika Penyelidikan (Manusia) USM
Pelantar Penyelidikan Sains Klinikal, USM Kampus Kesihatan.
No. Tel: 09-767 2355 / 09-767 2352
Email : jepem@kk.usm.my

KERAHSIAAN

Maklumat perubatan anda akan dirahsiakan oleh doktor dan kakitangan kajian. Ianya tidak akan dedahkan secara umum melainkan jika ia dikehendaki oleh undang-undang.

Data yang diperolehi dari kajian yang tidak mengenalpasti anda secara perseorangan mungkin akan diterbitkan untuk tujuan memberi pengetahuan baru.

Rekod perubatan anda yang asal mungkin akan dilihat oleh pihak penyelidik, Lembaga Etika kajian ini dan pihak berkuasa regulatori untuk tujuan mengesahkan prosedur dan/atau data kajian klinikal. Maklumat perubatan anda mungkin akan disimpan dalam komputer dan diproses dengannya.

Dengan menandatangani borang persetujuan ini, anda membenarkan penelitian rekod, penyimpanan maklumat dan pemindahan data seperti yang dihuraikan di atas.

TANDATANGAN

Untuk dimasukkan ke dalam kajian ini, anda atau wakil sah anda mesti menandatangani serta mencatatkan tarikh halaman tandatangan (Lihat contoh Borang Keizinan Pesakit di **LAMPIRAN G**).

Borang Keizinan Pesakit/ Subjek untuk *Sampel Genetik*
(Halaman Tandatangan)

Tajuk Kajian: Pembangunan Sistem Kemandulan Berkomputer Kepintaran Berdasarkan Analisis Sperma Motilitas

Nama Penyelidik: Prof. Madya Dr Nor Ashidi Mat Isa
Dr Mahaneem Mohamed
Tan Weng Chun

Untuk menyertai kajian ini, anda atau wakil sah anda mesti menandatangani mukasurat ini. Dengan menandatangani mukasurat ini, saya mengesahkan yang berikut:

- Saya telah membaca semua maklumat dalam Borang Maklumat dan Keizinan Pesakit ini **termasuk apa-apa maklumat berkaitan risiko yang ada dalam kajian** dan saya telah pun diberi masa yang mencukupi untuk mempertimbangkan maklumat tersebut.
- Semua soalan-soalan saya telah dijawab dengan memuaskan.
- Saya, secara sukarela, bersetuju menyertai kajian penyelidikan ini, mematuhi segala prosedur kajian dan memberi maklumat yang diperlukan kepada doktor, para jururawat dan juga kakitangan lain yang berkaitan apabila diminta.
- Saya boleh menamatkan penyertaan saya dalam kajian ini pada bila-bila masa.
- Saya telah pun menerima satu salinan Borang Maklumat dan Keizinan Pesakit untuk simpanan peribadi saya.

______________________	______________________
**Nama Pesakit** (Dicetak atau Ditaip)	**Nama Singkatan & No. Pesakit**
______________________	______________________
**No. Kad Pengenalan Pesakit** (Baru)	**No. K/P** (Lama)
______________________	______________________
**Tandatangan Pesakit** atau Wakil Sah	**Tarikh** (dd/MM/yy)<br>Masa (jika perlu)
______________________	______________________
**Nama & Tandatangan Individu** yang Mengendalikan Perbincangan Keizinan (Dicetak atau Ditaip)	**Tarikh** (dd/MM/yy)
______________________	______________________
**Nama Saksi dan Tandatangan**	**Tarikh** (dd/MM/yy)

<u>**Nota:**</u>
i) Lebihan sampel kajian ini akan dilupuskan dan tidak akan digunakan untuk tujuan lain kecuali setelah mendapat kebenaran daripada Jawatankuasa Etika Penyelidikan (Manusia), USM.
ii) Semua subjek/pesakit yang mengambil bahagian dalam projek penyelidikan ini <u>tidak dilindungi insuran</u>.

Borang Keizinan bagi Penerbitan Bahan yang berkaitan dengan Pesakit/ Subjek (Halaman Tandatangan)

Tajuk Kajian: Pembangunan Sistem Kemandulan Berkomputer Kepintaran Berdasarkan Analisis Sperma Motilitas

Nama Penyelidik: Prof. Madya Dr Nor Ashidi Mat Isa
Dr Mahaneem Mohamed
Tan Weng Chun

Untuk menyertai kajian ini, anda atau wakil sah anda mesti menandatangani mukasurat ini.

Dengan menandatangani mukasurat ini, saya memahami yang berikut:

- Bahan yang akan diterbitkan tanpa dilampirkan dengan nama saya dan setiap percubaan yang akan dibuat untuk memastikan ketanpanamaan saya. Saya memahami, walaubagaimanapun, ketanpanamaan yang sempurna tidak dapat dijamin. Kemungkinan sesiapa yang menjaga saya di hospital atau saudara dapat mengenali saya.
- Bahan yang akan diterbitkan dalam penerbitan mingguan/bulanan/dwibulanan/suku tahunan/dwi tahunan merupakan satu penyebaran yang luas dan tersebar ke seluruh dunia. Kebanyakan penerbitan ini akan tersebar kepada doktor-doktor dan juga bukan doktor termasuk ahli sains dan ahli jurnal.
- Bahan tersebut juga akan dilampirkan pada laman web jurnal di seluruh dunia. Sesetengah laman web ini bebas dikunjungi oleh semua orang.
- Bahan tersebut juga akan digunakan sebagai penerbitan tempatan dan disampaikan oleh ramai doktor dan ahli sains di seluruh dunia.
- Bahan tersebut juga akan digunakan sebagai penerbitan buku oleh penerbit jurnal.
- Bahan tersebut tidak akan digunakan untuk pengiklanan ataupun bahan untuk membungkus.

Saya juga memberi keizinan bahawa bahan tersebut boleh digunakan sebagai penerbitan lain yang diminta oleh penerbit dengan kriteria berikut:

- Bahan tersebut tidak akan digunakan untuk pengiklanan atau bahan untuk membungkus.
- Bahan tersebut tidak akan digunakan di luar konteks – contohnya: Gambar tidak akan digunakan untuk menggambarkan sesuatu artikel yang tidak berkaitan dengan subjek dalam foto tersebut.

______________________________ ______________________________

Nama Pesakit (Dicetak atau Ditaip) **Nama Singkatan** atau **No. Pesakit**

______________________ ______________ ______________________

No. Kad Pengenalan Pesakit T/tangan Pesakit **Tarikh** (dd/MM/yy)

______________________________ ______________________

Nama & Tandatangan Individu yang Mengendalikan Perbincangan Keizinan (Dicetak atau Ditaip) **Tarikh** (dd/MM/yy)

Nota: i) Semua subjek/pesakit yang mengambil bahagian dalam projek penyelidikan ini tidak dilindungi insuran.

RESEARCH INFORMATION

Research Title: Development Of Computational Intelligence Infertility System Based On Sperm Motility Analysis

Researcher's Name: Prof. Madya Dr Nor Ashidi Mat Isa
Dr Mahaneem Mohamed (MMC: 31287)
Tan Weng Chun

INTRODUCTION

You are invited to participate in a voluntary research study involving the use of semen. Research is needed to develop the computerized intelligence systems based on the analysis of infertility sperm motility. This research will take excess semen that will be discarded after a normal semen fluid analysis conducted in Cytology Laboratory USM. Sperm movement is recorded by using a video camera and a microscope. Before agreeing to participate in this research study, it is important that you read and understand this form. If you participate, you will receive a copy of this form to keep for your records.

Your participation in this study is expected to take up to 4 weeks. A total of 110 patients participated in this study.

PURPOSE OF THE STUDY

This study is to examine the problem of infertility with sperm count motility through a computerized system. System will give more accurate results about infertility compare to the manual analysis.

There is a possibility that the information collected during this study will be analyzed by the researchers in the future to assess sperm motility for research purposes or for medical or scientific purposes other than those currently proposed.

QUALIFICATION TO PARTICIPATE

Researcher in charge of this study or a research staff has discussed the requirements for participation in this study with you. It is important that you be honest with the doctor and staff about your health history. You should not participate in this study if you do not meet all eligibility requirements.

Some of the requirements to participate in this study are

- You have been suspected of having infertility problems.
- You must have sexual abstinence for at least 2 to 3 days

STUDY PROCEDURES

During your visit, if you agree to participate, you must obtain permission from the doctor to undergo semen analysis. Semen will be obtained from you and send it to a pathology lab for testing manually. The remaining semen will be used to lead research studies by recording the video to be analyzed in the future.

RISKS

If you participate, you may experience discomfort and inconvenience when deal with hospital staff. But, remember what you do is very much appreciated by us and it is required to assist in the study of infertility problems. Just a sample in excess of semen provided will be used for the study. No more samples should be supplied for review purposes. If any important new information found during this study that may affect your consent to continue to participate in this study, you will be notified as soon as possible.

REPORTING HEALTH EXPERIENCES.

If you suffer any injuries, bad effects, or any unusual health experience during this study, make sure you tell the nurse or doctorin charge that allows you to run semen analisys.

PARTICIPATION IN THE STUDY

Your participation in this study is entirely voluntary. You may refuse to participate in this study or you may end your participation at any time, without any penalty or loss of benefits to which you are otherwise entitled.

Your participation may also be terminated by the doctors involved in the study without your consent. If you stop participating in this study, doctors involved in the study or one of the staff will talk with you about any medical issue with respect to termination of your participation.

POSSIBLE BENEFITS [Benefit to Individual, Community, University]

This study will be provided at no cost to you. You may receive information about your health than physical examination and laboratory tests performed in this study. Results or information from this study is hoped, will benefit patients in the future. You will not receive any compensation for participating in this study.

QUESTIONS

If you have any question about this study or your rights, please contact;

<Dr. Mahaneem Mohamed> & <31287>
<Department Physiology>
<Pusat Pengajian Sains Perubatan>
<USM Health Campus>
<No Tel: 09-7676158>

If you have any questions regarding the Ethical Approval or any issue / problem related to this study, please contact;

Puan Mazlita Zainal Abidin
Secretary of Research Ethics Committee (Human) USM
Clinical Sciences Research Platform
USM Health Campus
Tel. No. : 09-767 2355 / 09-767 2352
Email : jepem@kk.usm.my

CONFIDENTIALITY

Your medical information will be kept confidential by the study doctor and staff and will not be made publicly available unless disclosure is required by law.

Data obtained from this study that does not identify you individually will be published for knowledge purposes.

Your original medical records may be reviewed by the researcher, the Ethical Review Board for this study, and regulatory authorities for the purpose of verifying clinical trial procedures and/or data. Your medical information may be held and processed on a computer.

By signing this consent form, you authorize the record review, information storage and data transfer described above.

SIGNATURES

To be entered into the study, you or a legal representative must sign and data the signature page **[ATTACHMENT G]**

Patient/ Subject Information and Consent Form
(Signature Page)

Research Title: Development Of Computational Intelligence Infertility System Based On Sperm Motility Analysis

Researcher's Name: Prof. Madya Dr Nor Ashidi Mat Isa
Dr Mahaneem Mohamed
Tan Weng Chun

To become a part this study, you or your legal representative must sign this page. By signing this page, I am confirming the following:

- I have read all of the information in this Patient Information and Consent Form **including any information regarding the risk in this study** and I have had time to think about it.
- All of my questions have been answered to my satisfaction.
- I voluntarily agree to be part of this research study, to follow the study procedures, and to provide necessary information to the doctor, nurses, or other staff members, as requested.
- I may freely choose to stop being a part of this study at anytime.
- I have received a copy of this Patient Information and Consent Form to keep for myself.

____
Patient Name (Print or type)

____
Patient Initials and **Number**

____
Patient I.C No. **(New)**

____
Patient I.C No. **(Old**)

____
Signature of patient or Legal Representative

____
Date (dd/MM/yy)
(Add time if applicable)

____
Name of Individual
conducting Consent Discussion (Print or Type)

____
Signature of Individual
Conducting Consent Discussion

____
Date (dd/MM/yy)

____
Name & Signature of Witness

____
Date (dd/MM/yy)

Note:
i) All subject/patients who are involved in this study will not be covered by insurance.
ii) Excess samples from this research will not be used for other reasons and will be destroyed with the consent from the Research Ethics Committee (Human), USM.

Patient's Material Publication Consent Form
Signature Page

Research Title: Development Of Computational Intelligence Infertility System Based On Sperm Motility Analysis

Researcher's Name: Prof. Madya Dr Nor Ashidi Mat Isa
Dr Mahaneem Mohamed
Tan Weng Chun

To become a part this study, you or your legal representative must sign this page.

By signing this page, I am confirming the following:

- I understood that my name will not appear on the materials published and there has been efforts to make sure that the privacy of my name is kept confidential although the confidentiality is not completely guaranteed due to unexpected circumstances.
- I have read the materials or general description of what the material contains and reviewed all photographs and figures in which I am included that could be published.
- I have been offered the opportunity to read the manuscript and to see all materials in which I am included, but have waived my right to do so.
- All the published materials will be shared among the medical practitioners, scientists and journalist world wide.
- The materials will also be used in local publications, book publications and accessed by many local and international doctors world wide.
- I hereby agree and allow the materials to be used in other publications required by other publishers with these conditions:
- The materials will not be used as advertisement purposes nor as packaging materials.
- The materials will not be used out of contex – i.e.: Sample pictures will not be used in an article which is unrelated subject to the picture.

Patient Name (Print or type)

Patient Initials or **Number**

Patient I.C No.

Patient's Signature

Date (dd/MM/yy)

Name and Signature of Individual
Conducting Consent Discussion

Date (dd/MM/yy)

Note: i) All subject/patients who are involved in this study will not be covered by insurance.
