# Supplementary material for: Automated Sperm Head Detection Using Intersecting Cortical Model Optimised by Particle Swarm Optimization
Source: PLoS One. 2016 Sep 15;11(9):e0162985. doi: 10.1371/journal.pone.0162985 (PMC5025108; doi:10.1371/journal.pone.0162985)
Supplement: S3 File — (PDF) [file pone.0162985.s003.pdf]

Data Collection Form

A written consent will be obtained from the patient who send his semen for seminal fluid analysis in Cytology Laboratory, USM. A drop of sample will be taken from the excess volume of semen for seminal fluid analysis. Motility of sperm (Movement and Trajectory) will be recorded by using a microscope attached with a charge couple device (CCD) camera. The details will be like below:

Date :

Time :

File no :

Person in charge :

No. of spermatozoa in a frame :

Duration of the video :

Manual Analysis Result by Human Expert

(Cytology Technologist) : % Motility
